# External Data Systems Enable Enhanced (and Sustainable) Fourier Transform Mass Spectrometry Imaging for Legacy Hybrid Linear Ion Trap–Orbitrap Platforms

**DOI:** 10.1021/jasms.4c00145

**Published:** 2024-07-20

**Authors:** Franklin E. Leach, Konstantin O. Nagornov, Anton N. Kozhinov, Yury O. Tsybin

**Affiliations:** † Department of Chemistry, University of Georgia, Athens, Georgia 30602, United States; ‡ Spectroswiss, 1015 Lausanne, Switzerland

**Keywords:** FTMS, imaging, ultrahigh resolution, external data acquisition and processing, sustainability

## Abstract

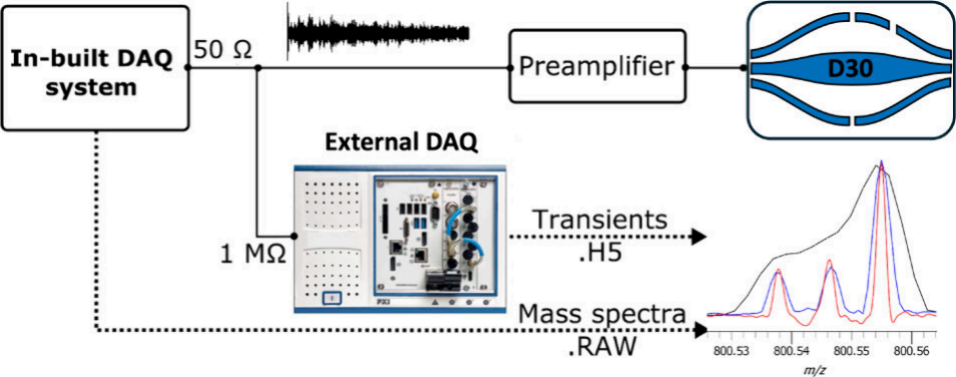

Legacy Fourier transform (FT) mass spectrometers provide robust platforms for bioanalytical mass spectrometry (MS) yet lack the most modern performance capabilities. For many laboratories, the routine investment in next generation instrumentation is cost prohibitive. Field-based upgrades provide a direct path to extend the usable lifespan of MS platforms which may be considered antiquated based on performance specifications at the time of manufacture. Here we demonstrate and evaluate the performance of a hybrid linear ion trap (LTQ)–Orbitrap mass spectrometer that has been enhanced via an external high-performance data acquisition and processing system to provide true absorption mode FT processing during an experimental acquisition. For the application to mass spectrometry imaging, several performance metrics have been improved including mass resolving power, mass accuracy, and dynamic range to provide an FTMS system comparable to current platforms. We also demonstrate, perhaps, the unexpected ability of these legacy platforms to detect usable time-domain signals up to 5 s in duration to achieve a mass resolving power 8× higher than the original platform specification.

## Introduction

Modern bioanalytical mass spectrometry (MS) strives to achieve the simultaneous acquisition of high-performance data with high experimental throughput. Compared to other MS platforms, Fourier transform mass spectrometry (FTMS) performance directly depends upon a linear increase in acquisition time domain to provide higher mass resolving power and mass accuracy for a given spectrometer generation. Within FTMS, two subsets exist, ion cyclotron resonance (ICR FTMS)^1^ and Orbitrap FTMS.^2^ Both rely on the observation of trapped ion frequencies to generate a time domain signal.

Typically, the most direct path to higher performance is to increase this experimental frequency so that more ion orbits are monitored per unit time. For ICR, the measured cyclotron frequency is directly proportional to the applied magnetic field, with current systems ranging from 7 to 21 T.^3,4^ For Orbitrap systems, the measured axial frequency is directly proportional to the square root of the applied electric potential and inversely proportional to the trap dimensions. Unfortunately, this improvement typically requires the purchase of a higher field magnet for ICR or a newer, complete Orbitrap system. Such purchases are typically cost-prohibitive for most analytical laboratories. On the other hand, field-based upgrades to a mass spectrometer can be achieved at lower cost to enhance the baseline analytical performance and therefore extend the lifetime of existing equipment. Such examples include aftermarket ion sources to provide matrix-assisted laser desorption ionization (MALDI) as an alternative to electrospray ionization (ESI),^5,6^ incorporation of laser and electron-generation systems for ionization or dissociation,^7−13^ and data acquisition and processing solutions.^14,15^

The suitability of each option depends on the target analytes and desired experimental end point. Mass spectrometry imaging (MSI)^16,17^ is an experimental approach that continues to gain interest among the scientific community due to the provision of both spatial and chemical information in a label-free manner. MSI allows for the localization of molecules from complex surfaces with the dominant application space in animal and plant tissues to elucidate spatial aspects of biology. Many MSI modalities now exist in the field. MALDI MSI^18^ remains the most widespread and has been used to spatially map neuropeptides,^19^ lipids,^20^ drugs and metabolites,^21^ glycans,^22^ and intact proteins^23^ from biological tissue sections with extremely high molecular specificity.

Most mass analyzers have been coupled with MALDI to achieve imaging platforms. ICR FTMS offers the highest mass resolving power and mass accuracy for MS imaging experiments. ICR FTMS imaging provides detailed molecular information but at the cost of extended measurement time when high spectral resolution and spatial resolution are required. Mass resolution improves linearly, while mass accuracy and dynamic range improve quadratically with increasing magnetic field. Thus, higher field ICR FTMS imaging provides more confident identification of more species. In addition, image acquisition time can be reduced at higher field, since high mass resolving power is achievable with shorter time domain acquisitions. This is especially important at high spatial resolution, where reducing the pixel raster size by 2-fold (e.g., 50 to 25 μm) results in 4-fold more mass spectra collected. For a given magnetic field strength, mass resolving power can be enhanced by the application of absorption mode signal processing where the phase of ion motion is taken into consideration to provide an approximately 2-fold increase in resolving power for a given acquisition period.^24,25^ Orbitrap systems have adapted this strategy on modern systems with a proprietary processing algorithm known as an enhanced Fourier transform (eFT).^26,27^

Historically, ICR has presented a more modular and readily modified platform for FTMS with respect to mass analyzer (cell) design and novel data acquisition and processing schemes. The Orbitrap in comparison is a precision-engineered platform that has dramatically expanded the accessibility of FTMS to nonspecialists but precludes many significant hardware alterations by the MS instrumentation community and provides limited availability to the true raw (unreduced) data format known as a transient. Data acquisition strategies though remain an opportunity for enhancements, especially for legacy Orbitrap systems such as linear ion trap (LTQ) hybrid platforms that still remain in service. Since these systems share a common signal detection scheme with ICR in the Fourier transformation of a time domain signal, the FTMS community has been successful in integrating data acquisition (DAQ) systems after signal preamplification with examples spanning application spaces including intact protein analysis,^28^ top-down proteomics,^29^ isotopic ratio analysis,^30^ environmental analysis,^31^ and MSI.^32,33^

Here we describe the enhanced analytical performance of legacy hybrid Orbitrap instrumentation (ca. 2005 LTQ Orbitrap XL) to meet the demands of modern biological mass spectrometry imaging (MSI). This platform predated the incorporation of the eFT signal processing and produces only data in magnitude mode.^34^ Although common in current offerings, this Orbitrap generation lacked advanced signal processing (eFT or absorption mode) and the higher applied field during ion detection to induce increased axial frequencies present in later releases. These platforms also rely on computational hardware with limited/fixed memory and processors. External data systems provide a direct path to modern computational performance. The ion signal can be readily split after the preamplifier and processed according to the desires of the end user. To this end, we have been able to acquire in-hardware phased time domain signals and process them with true absorption mode algorithms during an experiment. Previously this capability was only available by postprocessing of LTQ Orbitrap transients by software (e.g., Autophaser^15^) and presents a workflow bottleneck due to time demands. Moreover, the accessible transient periods were limited by the performance of the in-built DAQ system.

The incorporation of a high-performance DAQ system has enabled true absorption mode processing and extended duration transients which provides over 8-times the baseline performance as commercially available at the time of production. The achieved performance metrics described here rival that of more recent MS instrumentation at a fraction of the required cost and therefore provide an avenue for the MS community to readily improve systems still in daily use and further extend their lifetimes. At this time, widespread efforts have been made to reduce laboratory waste streams including plastics (e.g., pipet tips) and foam sample coolers, but the fate of instrumentation is not entirely known after a spectrometer has been retired. MS systems are complex, integrated ensembles of metals, plastics, ceramics, electronic circuit boards, and other materials that pose a challenge for traditional recycling approaches. Some are acquired by second-hand vendors and resold or parted out which reduces the materials entering waste streams. Our approach further extends both the performance and usable lifetime of existing equipment, therefore providing an opportunity to enhance the sustainability of mass spectrometry.

## Experimental Methods

### Materials

Chemical reagents (UPLC water, HPLC methanol, and 2,5-dihydroxybenzoic acid) were purchased from Sigma-Aldrich (St. Louis, MO).

### Sample Preparation

Murine brain (Jackson Laboratories) was cryosectioned on a CM1950 cryostat (Leica Biosystems, Nussloch, DE) at −20 °C and 14 μm thickness. Sections were thaw mounted onto ITO-coated slides (CG-90IN-S110; Delta Technologies, Loveland, CO). Mounted slides were archived at −80 °C until MSI sample preparation. MALDI matrix was applied with a TM Sprayer (HTX Technologies). DHB was prepared at 40 mg/mL in 70% aqueous methanol and applied with a flow rate of 0.05 mL/min, 16 passes with a criss-cross pattern, track spacing of 4 mm. The nebulizer nozzle was held at 70 °C, maintained at a height of 40 mm, and moved with a velocity of 1300 mm/min. A drying time of 10 s was added to the end of each pass. After completion of the matrix application, slides were placed in desiccator bell jar for 10 min to ensure complete drying before each MSI experiment.

### Mass Spectrometry

Experiments were conducted with an LTQ Orbitrap XL Fourier transform mass spectrometer^34^ (Thermo Fisher Scientific, Bremen, Germany) in positive ion mode. A dual ESI-MALDI ion source^5^ (Spectroglyph, Kennewick, WA) with dual ion funnels was used for all experiments with the low-pressure funnel maintained at 7.5 Torr. Prior to MSI, the instrument was calibrated with Pierce LTQ Velos positive ion calibration mix. Additional tuning of the ion path through the add-on ion source and mass spectrometer was performed with a calibrant peak at *m*/*z* 524.2650 due to its proximity to the mass range of interest for the experiments. The laser (λ = 355 nm; Explorer-349-060, Newport, Irvine, CA) was operated at 0.5–1 kHz. Automatic gain control (AGC) was disabled, and the imaging experiment was performed with a defined ion injection time on the front-end LTQ XL to produce pixels of equal time duration. The number of laser shots is proportional to the injection time, and between 175 and 350 laser shots were collected per position. Images were acquired with a stage raster ranging from 25 to 100 μm.

### Data Acquisition

A high-performance DAQ system (FTMS Booster X2, Spectroswiss, Lausanne, Switzerland) was inserted between the in-built DAQ system and instrument preamplifier. The in-built DAQ can still interface with the primary control computer for normal operation. The setup was employed for all MSI experiments performed in this work.

Orbitrap FTMS spectra were acquired with a specific mass resolution of 60 000 or 100 000 depending on the experiment. (Note: this metric is specified at *m*/*z* 400 in this generation whereas newer Orbitrap systems are typically based on *m*/*z* 200). MS data sets were acquired in parallel, both in the Thermo *.raw format as provided by the Orbitrap XL internal DAQ computer (magnitude mode FT, reduced profile mode) and in the *.h5 file format of the external DAQ and processing computer. Position coordinates for the MALDI injector were acquired as an *.xml file.

### Data Analysis

Qualitative data analysis was performed in Qual Browser (Thermo Fisher Scientific) for total ion current (TIC) chromatogram visualization and MS scan examination and in Image Insight (Spectroglyph, Kennewick, WA) for initial image evaluation. Primary data processing and analysis were performed in Peak-by-Peak Mozaic (Spectroswiss). For each experiment, Thermo *.raw files and the corresponding *.xml position file were converted to the *.h5 file format with a noise thresholding level of 0. FTMS Booster X2 transient files were imported and scan aligned with their respective *.raw and *.xml files and then converted to the *.h5 file format with noise thresholding level of 0. Transient signals were zero-filled twice and apodized with a semikaiser (half window) function to optimize peak shape and sensitivity. Pixel-based recalibration, comparison of vendor and external data set attributes, and visualization of MS images were all performed in Mozaic. Isotopic fine structure (IFS) distributions were calculated in Bruker Isotope Pattern.

## Results and Discussion

### External Data System Integration

**Figure 1:**
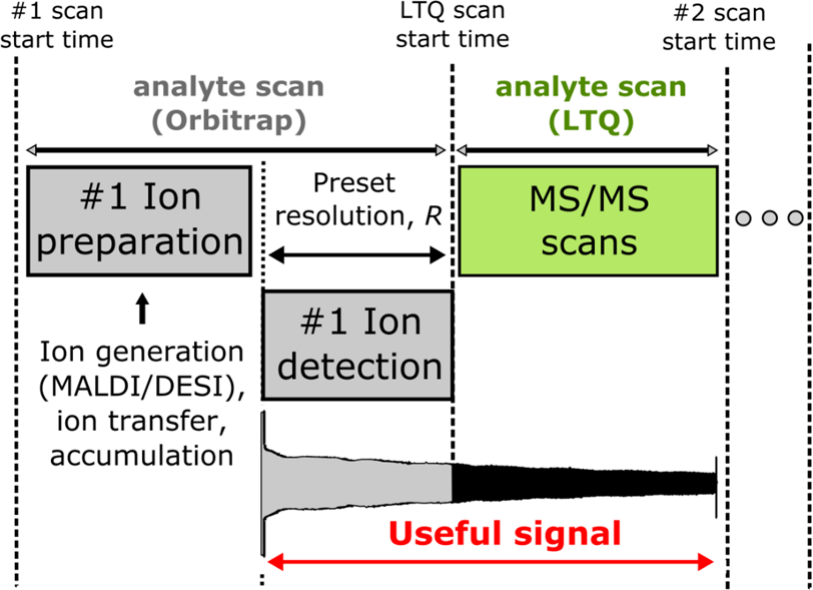
Experimental scheme to acquire phased transients on the modified linear ion trap Orbitrap system (LTQ Orbitrap XL, Thermo Fisher Scientific) with the integrated DAQ system. The useful signal can be extended by the incorporation of LTQ MS/MS scans in the experimental sequence.

The basis of our performance enhancements was the introduction of an external DAQ system to the ion detection circuitry of the LTQ Orbitrap XL to directly acquire and process the detected time domain signals after ion preparation shown in Figure 1. Following preamplification, the ion signal was split between the in-built DAQ system of the mass spectrometer to facilitate standard operation via the commercial control software and the external data system to provide phased transients.

A key distinction here is that a *.raw file from the instrument contains a magnitude mode mass spectrum whereas the external data system generates a *.h5 file that contains a phased transient for each acquisition that can be further processed into either a magnitude and/or absorption mode mass spectrum. The latter requires the acquisition of in-hardware phased transients, because upon injection into the Orbitrap mass analyzer from the C-trap, ions will have differing initial phases of motion.^35^ It is possible to determine the initial time point of coherence and provide a phase correction. Following this logic, the in-hardware phased transients are phase-corrected by real-time digital signal processing on the high-performance DAQ system. When these phase angles are close to zero, we can directly produce an absorption mode spectrum (half window apodization) where the appearance of peaks with negative intensity in the mass spectrum is minimized.

### Resolution Enhancements

When performed on a standard MS platform, MSI presents a significant mass resolution challenge due to the absence of any chromatographic separation which reduces the per scan spectral complexity during LC-MS. Ion mobility can be employed to perform a similar function in the gas phase after MALDI, but most MS systems lack this feature. Therefore, an MSI system should provide the highest possible mass resolving power. Common mass splits of 8.9 mDa (^13^C_2_ vs H_2_) and 2.4 mDa (^23^Na^1^H vs ^12^C_2_) occur in the range of *m*/*z* 600–950 for lipid MSI and require at least 50k to initially resolve an 8.9 mDa doublet with peaks of near equal intensity (∼1:1).^4,32,36,37^ This case would represent the lowest required resolving power for this doublet, with increased instrumental demand as this intensity difference increases. In samples where this intensity difference approaches 10:1, the MS imaging system must provide approximately 2× higher mass resolving power to confidently annotate the doublet.

Our work demonstrates the enhanced analytical performance of legacy hybrid Orbitrap instrumentation (ca. 2005 LTQ Orbitrap XL) to perform modern biological MSI. Specifically, we image the positive ion lipidome, which is a standard imaging experiment performed by many MS laboratories. Compared to current Orbitrap systems (Tribrid, Exactive, or Exploris lines), the stock LTQ Orbitrap XL provides a maximum selectable resolving power of 100 000 at *m*/*z* 400 (magnitude mode FT, 1.5 s transient).^34^ A defining aspect of Orbitrap performance is that frequency is directly proportional to the inverse of the square root of *m*/*z*. This relationship provides a distinct performance advantage at higher *m*/*z* compared to ICR.^38^ When compared to the nominal performance of a 7 T system, an inflection point occurs in the range of *m*/*z* 800 where a standard legacy Orbitrap outcompetes the ICR (both producing magnitude mode FT mass spectra).

**Figure 2:**
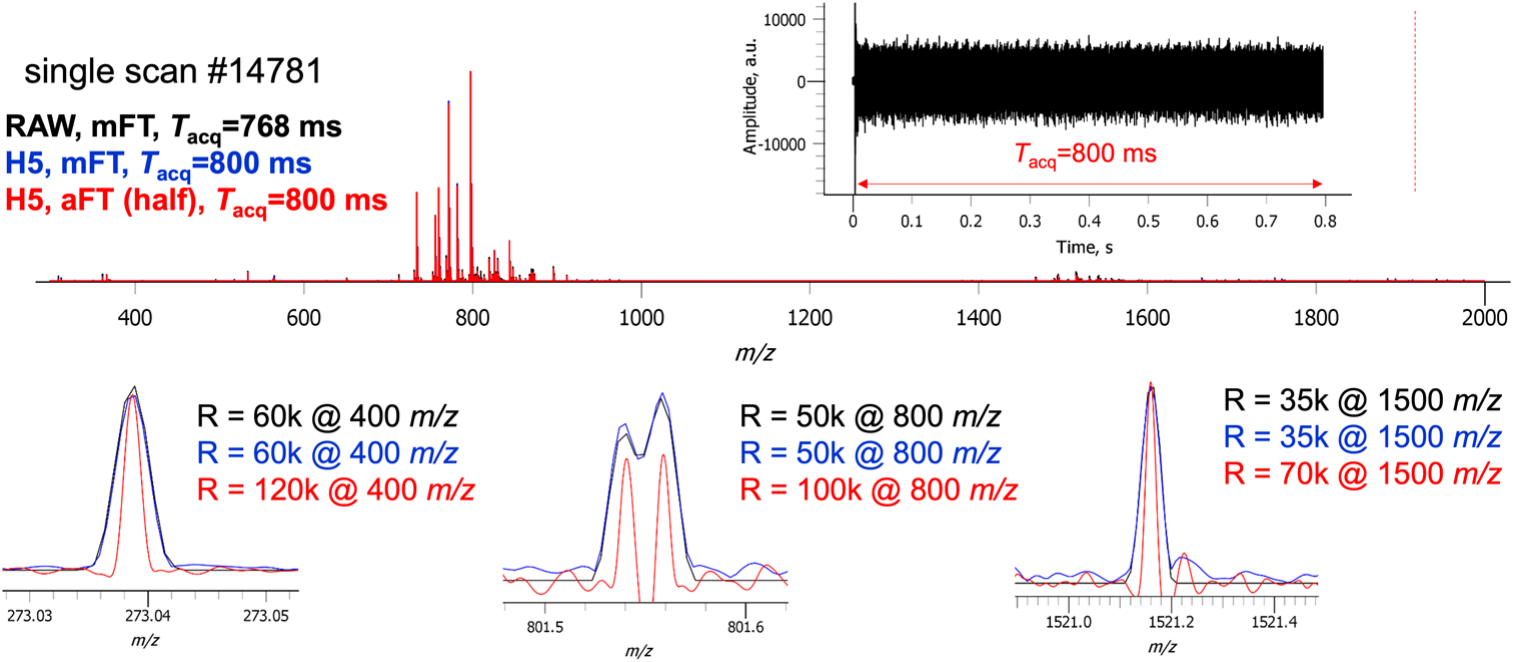
Performance comparison for a single scan (i.e., pixel) from a data set with a specified resolving power (RP) of 60 000 and experimentally measured resolution (*R*) performance. In comparison with magnitude mode FT (mFT), absorption mode (aFT) processing provides enhanced resolution across the mass range and enables increased confidence in the resolution of peak doublets.

Compared to a standard magnitude mode (mFT) spectrum, absorption mode FT (aFT) enables a 2-fold improvement in resolution across the entire mass range. Shown in Figure 2, we demonstrate this enhancement across our experimental *m*/*z* 200–2000 values. This data set was acquired on mouse brain with 2,5-dihydroxybenzoic acid (DHB) applied as the MALDI matrix to target lipids. In this instance, a moderate value of 60 000 at *m*/*z* 400 was specified in the vendor software to provide a 768 ms transient while external transients were acquired at a comparable 800 ms. The latter is a consequence of the differences between the architectures of the employed in-built and external DAQ systems. The in-built DAQ system acquires transients for the prespecified period, e.g., 768 ms. The external high-performance DAQ system acquires transients from the start trigger (e.g., ion injection into the mass analyzer) to the stop trigger (e.g., ion ejection from the mass analyzer). That signifies a principal ability of the employed high-performance DAQ system to perform trigger recognition using the advanced digital signal processing on the embedded field-programmable gate array (FPGA) chip in real time. Therefore, the 800 ms transient includes the 768 ms original transient and an overhead of 32 ms. For direct comparison and validation with the commercial *.raw files, external transients were also mFT mode processed. At low *m*/*z*, a DHB matrix ion provides an opportunity to examine this performance. Moving into the primary base peak region of the composite mass spectrum from *m*/*z* 700–900 corresponding to the ionized lipids, a doublet is barely resolved at *m*/*z* 801.57 and *m*/*z* 801.58 with a resolution of 50 000 in mFT mode. These features are then baseline resolved by provision of 100 000 in aFT mode. This 2× improvement is still achievable at *m*/*z* 1500 where we increase resolution from approximately 35 000 to 70 000.

**Figure 3:**
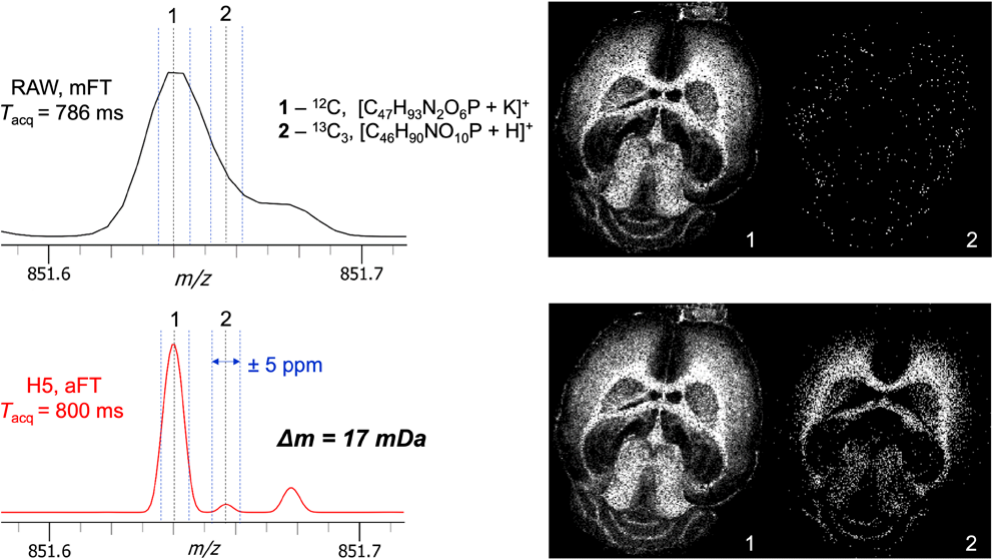
Two ions of interest with a spacing of 17 mDa are not resolved with the standard instrument mFT at 60 000 (upper left) and only the more intense peak with a defined apex can be visualized (upper right). With the provision of the aFT in the external DAQ (lower left), this doublet is well resolved and enables the generation of two distinct spatial distributions (lower right).

The benefits of aFT processing further enhance the ability to annotate lipid species in this data set. Examination of a mFT mode spectrum in the region from *m*/*z* 851.6–851.7 shows a peak with an unresolved shoulder at higher *m*/*z* (Figure 3).

Accurate mass measurement tentatively identifies the primary peak as the ^12^C of [C_47_H_93_N_2_O_6_P + K]^+^ without the ability to confidently identify an apex in the shoulder region. The generation of images within ±5 ppm produces a dominant image for the primary peak with a sparsely populated image for the apparent shoulder. Examination of the absorption mode spectrum reveals two baseline-resolved peaks and the opportunity to annotate the shoulder peak now as ^13^C_3_, [C_46_H_90_NO_10_P + H]^+^ with a well-defined image now visualized.

### Mass Accuracy Enhancements

**Figure 4:**
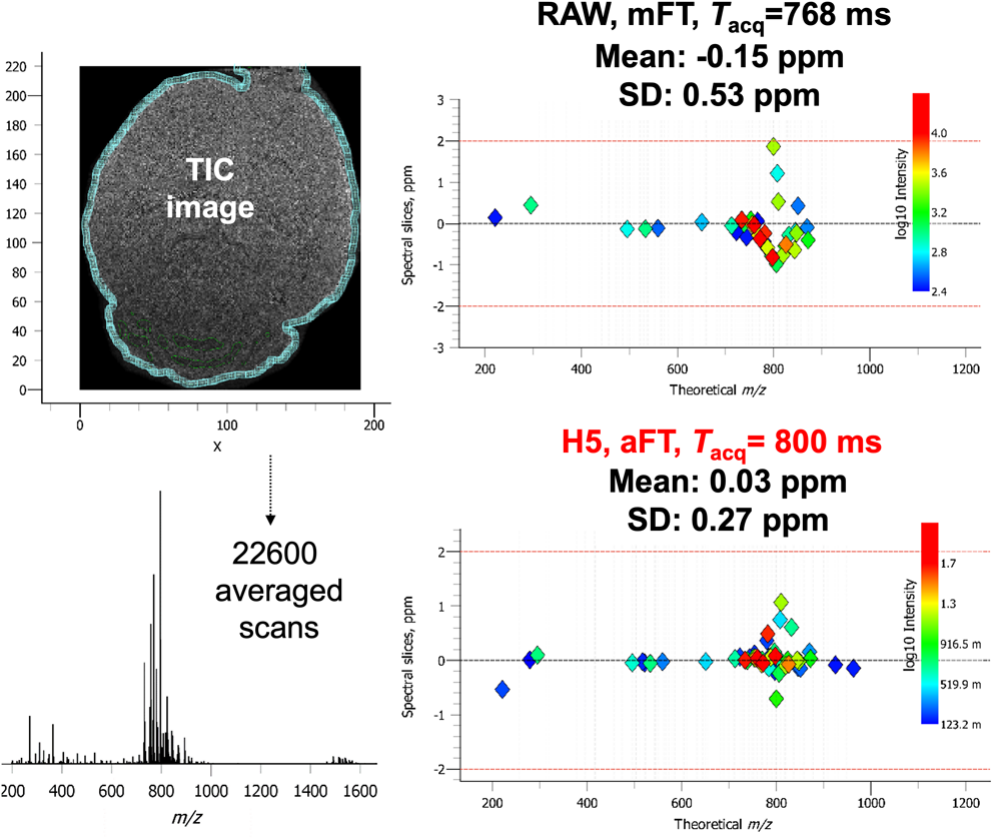
For a data set consisting of 22 600 pixels, the mass accuracies of selected peaks are improved in both overall mean error and standard deviation when the distribution of the raw mFT (upper right) is compared to that of the external data system aFT (lower right).

**Figure 5:**
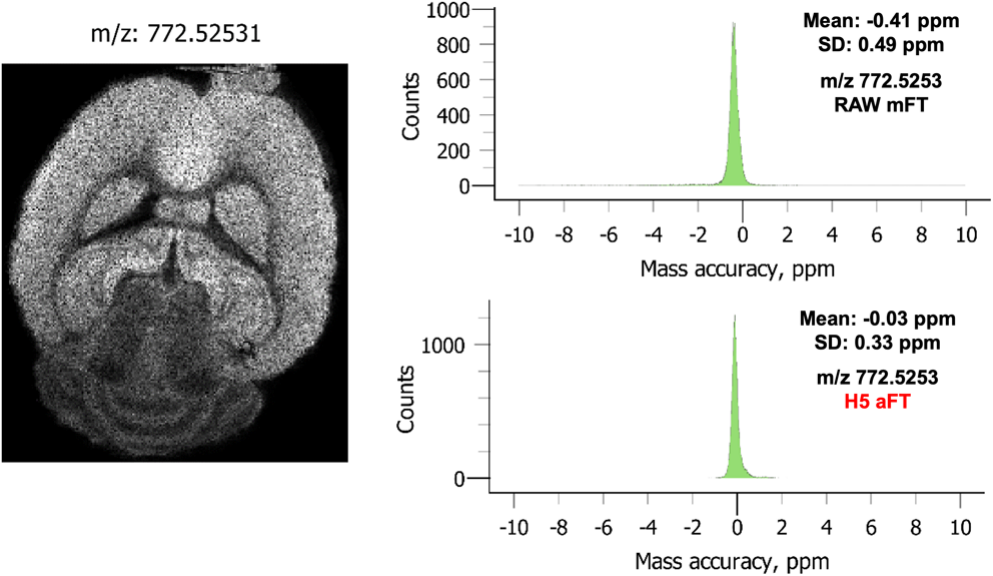
Pixel-based recalibration of a selected ion of interest results in over a 10-fold reduction in mean error with reduced standard deviation in the mass measurements.

A core challenge in MALDI MSI experiments is to acquire accurate mass measurement data with an ion packet that can vary from laser shot to shot.^39^ This difficulty is compounded when repeated over the thousands of pixel spectra from an MSI experiment. Returning to the composite mass spectrum from the 22 600 scans of this brain data set, the overall mass accuracy of all pixels can be evaluated. In Figure 4, the mass accuracies of reference compounds are shown from *m*/*z* 200–1200. In these data, only modest improvements are made at low *m*/*z* where the mass resolving power in FTMS is the highest combined with the sparse features. Overall, the absolute mean error is reduced 5-fold when aFT mode is enabled. This reduction directly follows from the resolution improvements that provide a well-defined apex on which to assign a *m*/*z* value. The most significant changes are observed at higher *m*/*z* where most of the lipid species are detected, again based on the ability to resolve more features with absorption mode processing of the transients. Additional recalibration on an individual pixel basis further reveals the benefit of phased transients on this legacy system shown in Figure 5. Demonstrated for an ion of interest at *m*/*z* 772.525, the mFT mode provides a mean error of −0.41 ppm across the image whereas the absorption mode provides nearly 14 times improved mean error of −0.03 ppm.

Similar to our prior example of resolving power improvements to lipid annotation MS imaging, the mass accuracy enhancements can also benefit annotation in these data sets. In this case for a specified 60 000 mFT resolving power on the Orbitrap XL (120 000 with aFT), we observe an average mass measurement error of 1.8 ppm reduced to 1.2 ppm with the data system upgrade. As an initial test of these enhancements on lipid composition determination, we converted our data set to the imzML format and performed a database search with an FDR of 10% in Metaspace.^40^ With the SwissLipids database, we observe an increase in putative identifications from 45 to 114 species and with HMDB-v4 an increase from 76 to 128 putative identifications.

### Ultrahigh Resolution Measurements

Standard end users are restricted to the hardware and software limitations of a given MS platform. For the Orbitrap XL platform, a specified resolution of 100 000 at *m*/*z* 400 is the highest level of performance under standard operation conditions with a transient duration of 1536 ms. Based on our prior discussion, this metric can be doubled to 200 000 by provision of phased transients and aFT mode spectra without any additional instrument time allocated per pixel. To further extend performance, one must increase the time period over which the time domain signal is generated and detected.

Our evaluation also serendipitously revealed the ability to acquire transients beyond this time limit up to 5 s in this instrument generation because the detection circuitry is not explicitly coupled to a resolution setting. By developing an instrument method that includes “dummy” scans in the LTQ XL, the experimental sequence can be extended beyond the maximum time for a given resolution setting, shown previously in Figure 1. With the addition of the external DAQ system, we can continually acquire data until the specified LTQ scans are complete. A key aspect of the employed external DAQ system is that the detection period does not have to be preset nor equal to a power of 2 in the total number of data points, as needed in previous (and contemporary) FTMS systems. Naturally, the central electrode potential on the spindle electrode of the Orbitrap mass analyzer should remain during the whole ion detection period. Therefore, the 5 s limit was selected to avoid potential system overheating for longer ion detection events. Recently, this approach allowed the acquisition of up to 10 s transients on the state-of-the-art Q Exactive HF^32^ and 24 s transients on the Q Exactive UHMR platforms.^41^ For example, the 10 s transients acquired from Q Exactive HF Orbitrap system enabled mass resolutions over 1 000 000 in the lipid *m*/*z* range (600–950 *m*/*z*) during MSI.^32^

**Figure 6:**
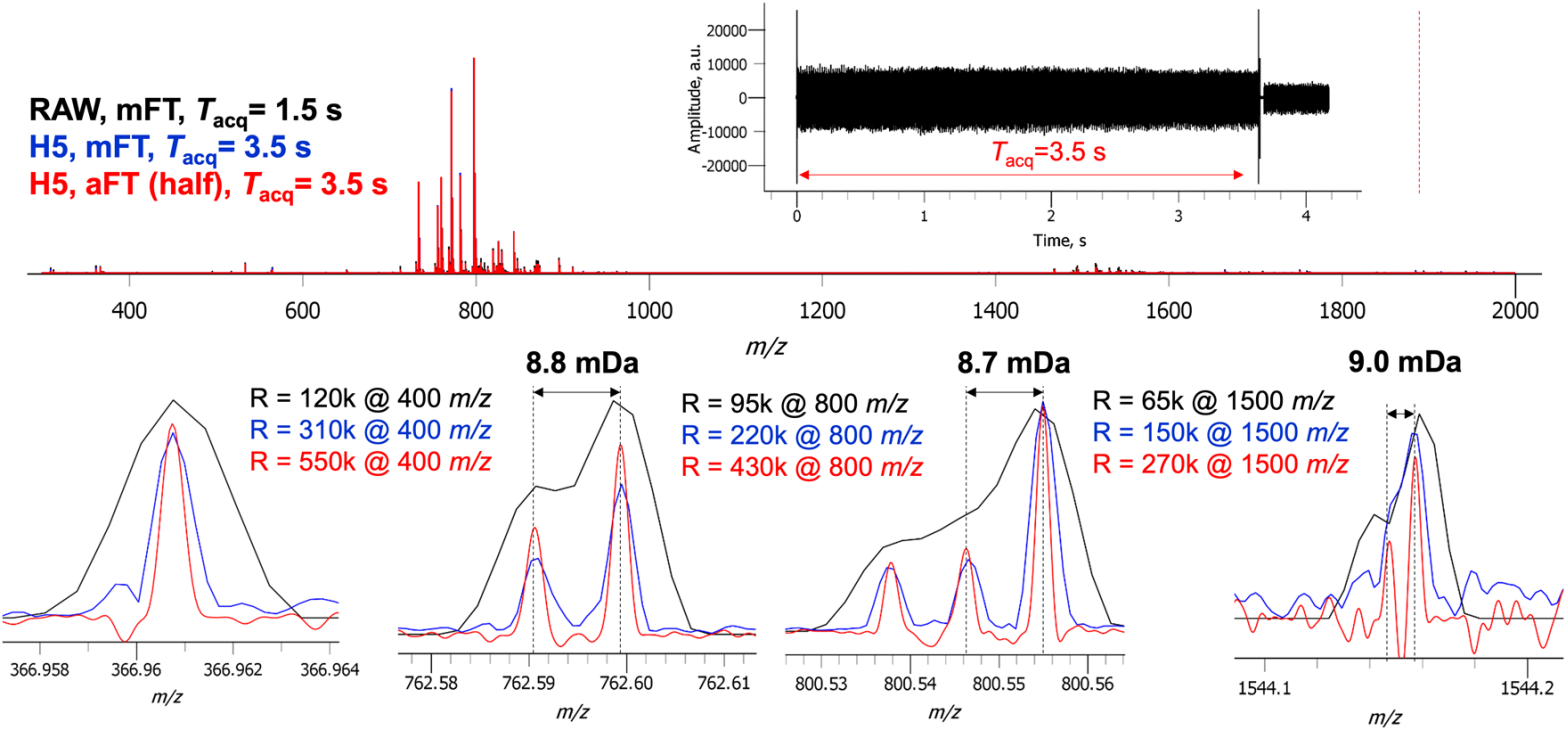
A single MS scan from an image acquired with extended transients reveals the potential for further enhancements in performance. An increase of 2 s beyond that of the highest possible resolution setting provides 4.2–4.5× the resolving power across the mass range.

To evaluate the performance of this LTQ Orbitrap XL operation in extended transient mode, we first opted for a 2 s increase in the transient length for a full image. Although one can acquire data for longer time periods, there is potential harm to the detection electronics, especially when performed repeatedly over the thousands of pixels and multiple hours required to generate a complete tissue image. With our 3.5 s acquisition time, we observe a substantial increase in resolution across the entire mass range, Figure 6. Compared to the standard operation at 1.5 s and 100 000 in mFT, the 3.5 s aFT enables a mass resolution of approximately 550 000 at *m*/*z* 366.96, an increase of 4.6×. This performance slightly decreases with increasing *m*/*z* but still results in the ability to achieve the resolution of mass doublets near 8–9 mDa from *m*/*z* 700–1500 whereas in the normal operating mode data, these features are not resolved.

**Figure 7:**
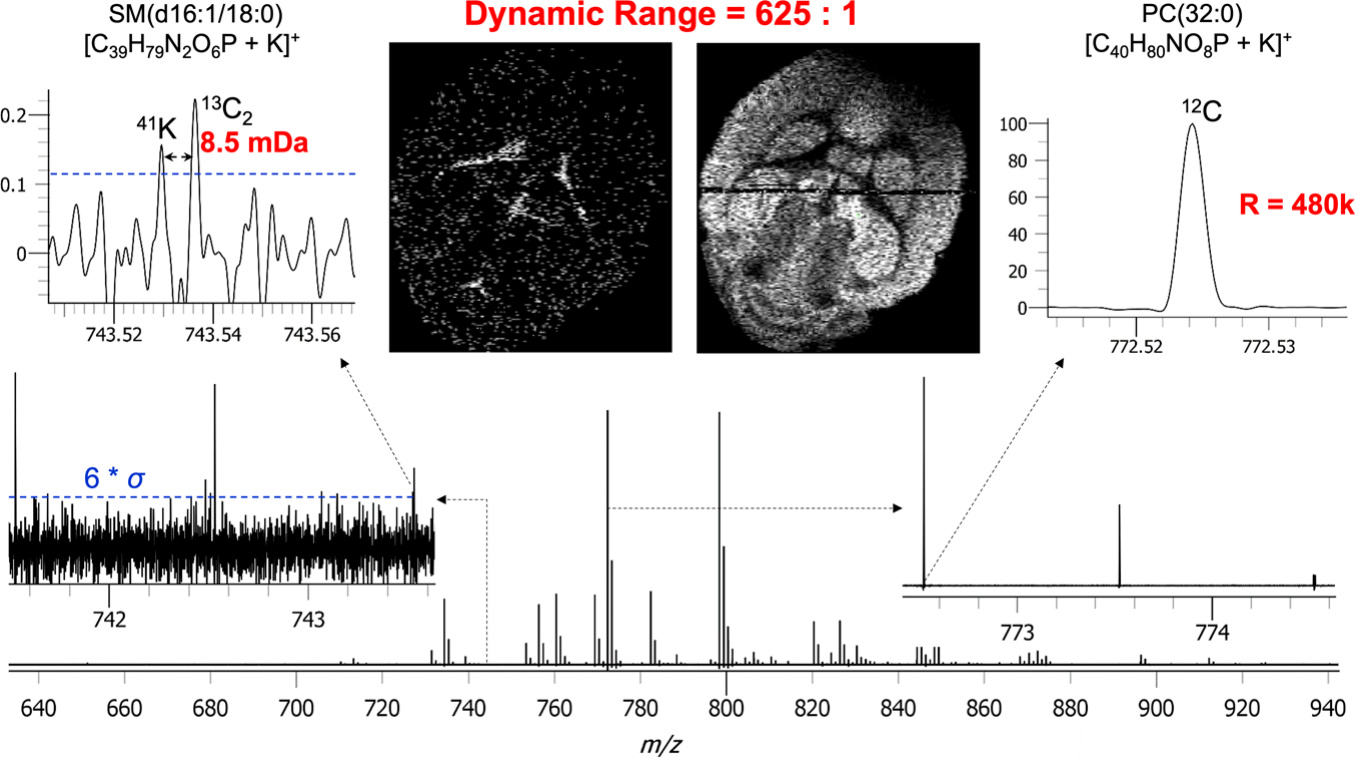
Demonstration of the potential for high dynamic range over an MS image acquired with extended transients on the Orbitrap XL MSI platform. The dynamic range between the base peak, PC(32:0), and lowest intensity doublet are depicted.

The acquisition of longer transients to provide ultrahigh resolution measurements also enables an increase in sensitivity for these MSI experiments. Whereas resolution will increase linearly in this case, sensitivity will increase as a square root of the transient length, both in the absence of signal decay. From our extended transient data set, we were able to leverage both of these enhancements to provide a dynamic range comparable of that provided by the state-of-the-art MALDI MSI performed on the 21 T FT-ICR at the National High Magnetic Field Laboratory.^4^ As shown in Figure 7 for a data set peak-picked at 6σ, our base peak occurred at *m*/*z* 772.52 with a signal-to-noise ratio (S/N) of 625 and resolution of 480 000 and was annotated as PC(32:0).

Our lowest intensity assignment was made by the resolution of an 8.5 mDa doublet and was putatively annotated as SM(d34:1) just above our S/N threshold indicated by the dashed line. This measurement resulted in a dynamic range of ∼625:1. Although we have not provided comparable resolution to the 21 T system (∼875 000 at *m*/*z* 800 vs ∼450 000 at *m*/*z* 800 here), we nonetheless achieve comparable dynamic range to their achievement of 536:1, indicating the performance potential of this legacy system.

**Figure 8:**
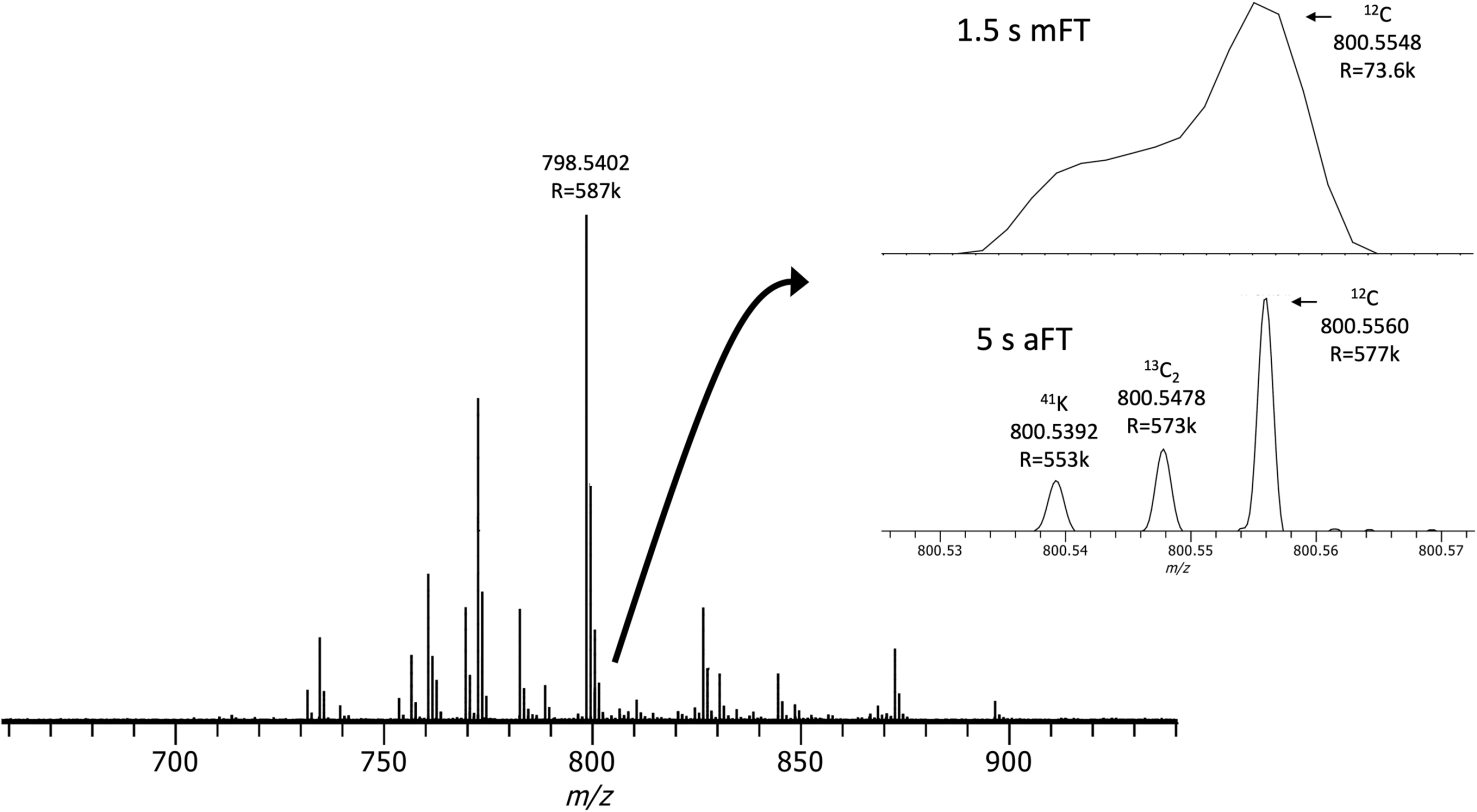
Acquisition of 5 s transients for lipid MALDI on tissue enables the resolution of a peak triplet that can be attributed to the combination of overlapping isotopic distributions that differ by a double bond and IFS features.

Although we did not acquire a complete image with a 5 s transient per pixel, we did acquire MALDI mass spectra from mouse brain in this mode. As expected, we observed further increases in the resolution provided by this platform with our average value near 600 000 in the lipid mass range. Shown in Figure 8 is a triplet that arises from the combination of overlapping isotopic distributions in lipid MS (^13^C_2_ vs H_2_) and isotopic fine structure (IFS). This base peak ion at *m*/*z* 798.540 is putatively assigned as PC(34:1)+K^+^. The standard maximum resolution setting of 100 000 (1.5 s transient) with mFT mode cannot provide the performance to resolve these features in the A+2 region near *m*/*z* 800.55 whereas a 5 s transient with aFT processing can baseline resolve them.

The right doublet can be assigned to the mass difference between the ^13^C_2_ peak of PC(34:1)+K^+^ (observed *m*/*z* 800.5478, theoretical *m*/*z* 800.547685, mass error −0.13 ppm) and the other to the ^12^C peak of PC(34:0) (observed *m*/*z* 800.5556, theoretical *m*/*z* 800.555614, mass error 0.83 ppm) which differs in one degree of unsaturation. The far-left peak at *m*/*z* 800.5392 can be assigned to the ^41^K isotopologue of PC(34:1)+K^+^ (theoretical *m*/*z* 800.539082, mass error −0.20 ppm). The natural abundance of the primary stable isotope of potassium, ^39^K, is 93.26% and that of ^41^K is 6.73% which makes this ^41^K isotopologue a key feature of the IFS profile for this ion with theoretical relative abundance of 7.3%. When compared to the theoretical relative abundance of the ^13^C_2_ peak of 10.2%, our single scan measurement is in general agreement with a peak ratio of ∼80% compared to a theoretical of 70% given the absence of better ion statistics that would be provided by averaging multiple scans. Averaging is not a common practice in MSI where a pixel is typically represented by a single scan. Nonetheless, this IFS measurement provides additional annotation confidence and illustrates the higher end of instrumental performance capable on this system.

## Conclusions

We have demonstrated the ability of a legacy hybrid linear ion trap Orbitrap platform to perform in the regime of modern MALDI FTMS equipment by the addition of an external high-performance DAQ system. This add-on requires no hardware modification to the instrument and opens up a realm of resolution and mass accuracy that could be seen as out of reach by some laboratories. Here we have illustrated this capability for MALDI-based mass spectrometry imaging (MSI). The incorporation of similar data acquisition schemes along with MALDI can be seen as a low-cost option for investigators to repurpose legacy spectrometers as a means to explore MSI without significant investments in new equipment and at a fraction of the cost. Although this system contains a low-field Orbitrap (D30 geometry and 3.5 kV central electrode potential) and therefore requires the acquisition of longer transients (3.5–5 s) for ultrahigh resolution performance due to lower measured axial frequencies, these experiments are typically performed on targeted sections of a tissue for MSI and not entire samples which would be measured under our standard operating conditions demonstrated here with 786 ms transients. Images can be acquired for most tissue sizes in several hours with the required resolution to identify ions of varying unsaturation, a key feature of lipid MSI. Although we observe performance enhancements here on this specific LTQ Orbitrap XL, these outcomes are dependent on the manufacturing quality of a particular system and may vary between platforms.

## Data Availability

The data underlying this study are openly available in Metaspace at https://www.metaspace2020.eu/project/Orbitrap_XL_w_External_DAQ_2024. Both *.raw and *.h5 data sets have been converted and are available in the *.imzml format.
